# Current Attitudes and Barriers Among Appalachian Patients Towards Orthopaedic Surgery

**DOI:** 10.13023/jah.0604.06

**Published:** 2025-01-29

**Authors:** Dylan Smith, Justin B. West, Wade Smith, Micah MacAskill, Jonathan Lash, Matthew W. Bullock

**Affiliations:** Penn State University Department of Orthopaedic Surgery; Marshall University Joan C. Edwards School of Medicine; Marshall University Department of Orthopaedic Surgery; Marshall University Department of Orthopaedic Surgery

**Keywords:** Appalachia, geographic barriers, medically underserved, orthopaedic access, rural healthcare, socioeconomic factors

## Abstract

**Introduction:**

This study explores the challenges faced by residents of rural West Virginia in accessing orthopaedic care due to geographic and socioeconomic barriers.

**Purpose:**

This study seeks to survey rural West Virginia residents about their attitudes and experiences regarding access to orthopaedic care in southern West Virginia.

**Methods:**

A total of 132 electronic surveys were collected from rural West Virginia residents during an appointment with their primary care provider located at an affiliated outreach clinic. The survey was designed to investigate various factors influencing their access to orthopaedic care.

**Results:**

Delays in seeking orthpaedic care are common in rural West Virginia. A shortage of orthopaedic specialists and the long distances required to travel to treatment centers significantly exacerbates health issues and complicates the management of orthopaedic conditions. Although participants express high levels of satisfaction with their orthopaedic treatment, they consistently identify the remote location of specialized services as the primary barrier.

**Implications:**

The study highlights the need for further research to evaluate the feasibility of expanding orthopaedic services into more isolated regions of West Virginia. This approach could improve healthcare access and potentially lead to better orthopaedic outcomes for these underserved populations.

## INTRODUCTION

Seventy-five percent of West Virginia’s (WV) citizens reside in counties designated as Health Professional Shortage Areas (HPSAs), making access to quality orthopaedic care particularly challenging for rural communities.^1^ Limited resources and various social barriers – including transportation challenges, lack of health education, and insufficient healthcare infrastructure – can delay patients’ presentation to orthopaedic clinics.^2^ Such barriers are uniquely exacerbated in WV due to its mountainous geography, sparse population, and underdeveloped healthcare infrastructure. Rural populations, which make up most of WV, often experience higher rates of morbidity and mortality compared to their urban counterparts.^3^ Additionally, rural patients are less likely to seek general medical care, which can result in delayed treatment for specialized needs like orthopaedic conditions.^4^ Such delays in presentation allow patient morbidities to progress, potentially complicating surgical treatment.^5^

West Virginia is the only state entirely within the Appalachian Region, an area Congress targeted in 1965 with the establishment of the Appalachian Regional Commission (ARC) to address socioeconomic underdevelopment.^6^ Despite improvements, the region still ranks among the lowest in the United States regarding socioeconomic status.^6^ West Virginia, with a population of just over 1.7 million, has a high prevalence of obesity, low median income, and the lowest rate of higher education attainment in the nation.^7^,^8^ These socioeconomic factors, coupled with high rates of orthopaedic-related conditions such as osteoarthritis and traumatic injuries, underscore the significant healthcare disparities within the state.

Orthopaedic care in WV is limited, particularly in rural areas where access to specialized services is scarce. Many regions of the state lack local orthopaedic surgeons, forcing residents to travel long distances to receive care, which can delay treatment and exacerbate conditions. The shortage of providers, compounded by geographic isolation, creates significant barriers for individuals in need of orthopaedic services, especially those with limited transportation options. As a result, West Virginians may experience increased pain, reduced mobility, and a lower quality of life, further underscoring the urgent need for expanded access to orthopaedic care in underserved areas of the state. Compared to more populated states, WV continues to face challenges with the density of orthopaedic surgeons and advanced practice providers.^9^,^10^

The specific counties in this study — Lincoln, Logan, McDowell, Wayne, and Wyoming — are among the most socioeconomically depressed in WV.^10^ These counties share high poverty rates, low educational attainment, and limited access to healthcare resources, mirroring challenges faced across the Appalachian Region. Accessing healthcare in these counties often requires navigating challenging terrain and self-reported long travel times, further deterring residents from seeking timely medical attention.

Barriers to orthopaedic care include not only geographic isolation and transportation challenges but also the shortage of orthopaedic specialists.^11^ Most specialists are concentrated in urban WV hubs such as Charleston, Huntington, and Morgantown.^12^ Rural clinics often lack modern facilities or specialized surgical equipment, making it difficult to provide comprehensive care. Delayed presentation for orthopaedic conditions often leads to advanced arthritic disease that may be more difficult to manage and may result in worse outcomes.^13^ To our knowledge, no studies have investigated access to orthopaedic care in WV. To better understand these barriers and inform potential solutions, this study aims to explore patient perspectives on accessing orthopaedic care in rural WV.

## METHODS

### Study Design

**Figure 1:**
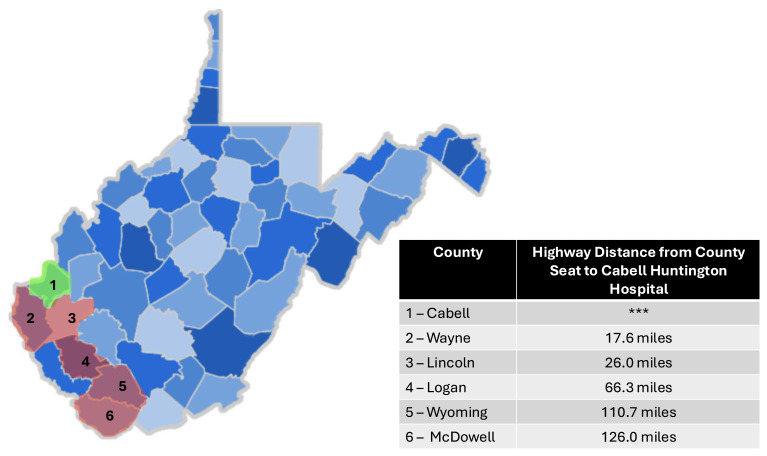
Location of the Main Hospital Institution and Distance in Miles to Rural Counties NOTE: Outreach clinics are located in Lincoln and Logan Counties.

This study employed a cross-sectional mixed-methods design, combining quantitative surveys and qualitative open-ended questions to investigate factors influencing orthopaedic care access. Data were collected between June 2021 – June 2022 using the REDCap survey platform to ensure efficient and secure data management. The survey design included demographic questions, Likert scale items, and open-ended questions to capture both numerical data and nuanced patient experiences. The target population comprised orthopaedic surgery patients residing in five southern WV counties (Lincoln, Logan, McDowell, Wayne, and Wyoming), all designated HPSAs **(**Fig. 1).

### Participant Recruitment

To recruit participants, rural healthcare providers (physicians and nurses) affiliated with the hospital network invited patients to participate during their primary care visits. Inclusion criteria encompassed all patients willing to participate. Surveys were anonymous, ensuring confidentiality and encouraging honest responses. Each participant received a $25 gift card as an incentive. No pilot testing was conducted for non-validated instruments, and existing validated tools had not been previously tested in rural or underserved populations. Funding for this study covered up to 150 survey responses.

### Survey Instruments

**Figure 2:**
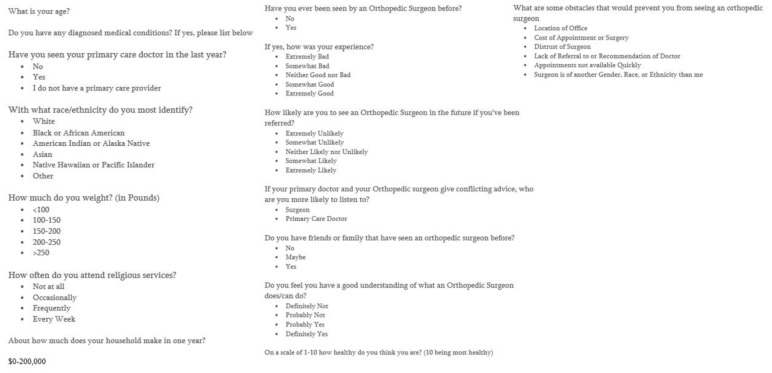
Survey

The survey featured several scales, including Likert scales to measure satisfaction with past orthopaedic care, likelihood of seeking future care, and perceived barriers to access. Questions addressed demographic factors such as age, income, educational attainment, and health status, as well as variables relevant to orthopaedic care, including transportation availability, previous treatment experiences, and proximity to care centers. Other questions, which allowed participants to select multiple answers, enabled them to elaborate on their challenges and facilitators in accessing care. In total the survey took less than ten minutes to complete **(**Fig. 2).

### Ethical Considerations

The study received approval from the Marshall Health institutional review board. Surveys were designed to minimize bias with clear concise questions. The research team members reviewed responses to ensure survey completion. Ethical guidelines were followed throughout the data collection process.

## RESULTS

An 88% response rate (132/150) was achieved from the two outreach clinics participating in the study, which cover five rural WV counties. Demographic data are summarized in Table 1. The average age of participants was 57.7 years, and nearly all (99.2%) identified as white. Most respondents (98%) had seen their primary care physician within the past year, and 98% had previously consulted an orthopaedic surgeon. Regarding religious attendance, 76.3% of respondents attended church frequently, occasionally, or weekly, while 23.7% did not attend. Regarding weight, 61.9% of respondents were over 200 pounds, and the mean household income was $44,800. Participants rated their experiences with their orthopaedic surgeon very positively, with an average rating of 4.56 stars out of 5. When asked how likely they were to keep an orthopaedic appointment when referred by their primary care physician (PCP), they gave an average rating of 4.78 out of 5. Interestingly, the majority of respondents indicated they would trust the medical advice of their orthopaedic surgeon over that of their PCP. Overall, respondents were satisfied with their orthopaedic care, were likely to seek an appointment when needed, and trusted their surgeon’s advice. However, barriers to care remain. Sixty-one percent of respondents cited location as a reason for not seeing an orthopaedic surgeon, 17.8% cited cost, 3.4% cited distrust, and 45.8% mentioned lack of timely appointments. None of the respondents cited the race or gender of the surgeon as a reason to avoid orthopaedic care.

## DISCUSSION

This study aimed to identify and characterize the attitudes of rural WV patients toward orthopaedic care and to gain insights into how to improve this care. These findings provide a clearer picture of an orthopaedic patient seeking treatment: most respondents cited the office location as a barrier, and nearly half reported difficulty obtaining a timely appointment. Addressing these concerns could be significantly improved by increasing the number of satellite orthopaedic offices in remote areas and establishing periodic outreach clinics with a surgeon or a physician extender. However, implementing these solutions would take time. Additionally, it remains uncertain whether the patient volume in these remote counties would be sufficient to sustain a busy orthopaedic practice.

West Virginia includes a large, economically depressed region with areas that are physically difficult to access. Many residents live in remote locations with limited telecommunication, often restricted to standard phone lines. Further research is needed to evaluate the feasibility of expanding services to these areas. Alternatively, implementing a transportation service could improve access for patients in hard-to-reach locations or those facing long travel times.

Encouragingly, most patients surveyed expressed high satisfaction with their orthopaedic care, and only 3.4% cited distrust of physicians or the medical system as a reason for avoiding care. This suggests that access issues, rather than attitudes toward healthcare providers, are the primary barriers to receiving care. However, selection bias may influence these results, as the surveyed patients were contacted at their PCP office – as such, patients who are suspicious of medical providers may be missed by a survey administered in this manner.

Regarding confounding variables, our study focused on only five of the 48 counties in WV designated as HPSAs. These counties are adjacent to the medically better-served areas of Cabell, Putnam, and Kanawha, where most medical services in southern West Virginia are concentrated. Depending on patient location, the commute can be short, but it often involves traveling on two-lane roads from rural areas which can become treacherous during winter months. To gain a more comprehensive understanding of rural healthcare challenges, future research should include patients from the more mountainous and fragmented terrain of the state’s southern regions.

Rural orthopaedic clinics have been proposed to improve access to orthopaedic care. These clinics could address some acute conditions, such as simple fractures and nonoperative osteoarthritis management, but patients would still need to travel to facilities with specialized orthopaedic surgeons if surgery were required. Telehealth has proven beneficial in remote areas, mainly for routine follow-up visits and therapy-directed rehabilitation, but cellular networks and internet access in remote areas of WV are still an issue.^14^,^15^

In rural areas, many individuals lack health insurance and struggle to afford out-of-pocket expenses for surgical care. While charity care plays a vital role in providing access to necessary services, it can impose a significant financial burden on already struggling rural hospitals. However, our research revealed that only 17.8% of respondents identified cost as a barrier to orthopaedic care, suggesting that financial concerns may be less of an obstacle than expected in this specialty. This finding highlights the potential to alleviate some of the stress on hospitals that are provide charity care. Although governmental grants and programs exist to help offset these costs, securing such funding has become increasingly challenging.^16^

This study adds to the growing body of evidence on healthcare challenges in rural areas. Similar findings have been reported in studies on primary care access, which highlight the difficulty of recruiting and retaining providers in remote regions. Studies by Xue et al. and others underscore how barriers such as geographic isolation, limited infrastructure, and socioeconomic disparities compound access issues in underserved areas.^17^–^19^ Addressing these challenges requires coordinated efforts, including expanding telehealth, improving transportation networks, and incentivizing providers to practice in rural settings.^11^ The consistency of these findings across specialties highlights the importance of targeted policy interventions to reduce disparities in healthcare access.

### Limitations

This study has several limitations, as illustrated in Table 2, some of which reflect the challenges of reaching hard-to-access patients. The survey participants were drawn from individuals already attending a doctor’s office. These patients were within a reasonable travel distance of their PCP and were actively seeking care. Most had seen their PCP within the past year, suggesting a high level of health engagement or monitoring. Additionally, these participants were either established patients or had scheduled appointments, which likely facilitated their participation and reduced the burden of scheduling additional appointments. Selection bias may be a concern if dissatisfied individuals chose not to participate in the survey. Furthermore, the sample size of 132 respondents is relatively small, which may limit the generalizability of the findings.

## CONCLUSION

Overall, most respondents expressed high satisfaction with their orthopaedic care. Notably, the majority identified location, rather than financial constraints, as the primary barrier to accessing orthopaedic care. Despite this challenge, respondents demonstrated a relatively high likelihood of attending their appointments, suggesting that patients prioritize their care and find ways to overcome barriers, even when faced with significant travel distances. These findings underscore the need for further exploration of outreach clinics – whether through telemedicine or other innovative solutions – to better address the orthopaedic needs of rural patients who struggle to commute to larger hospital centers for specialized care.

**SUMMARY BOX**

**What is already known about this topic?**

Rural patients face limited resources and various social barriers, including transportation challenges, lack of health education, and insufficient healthcare infrastructure, all of which can delay their access to proper healthcare.

**What is added by this report?**

Most rural West Virginia residents are satisfied with their orthopaedic care; however, the majority identified the location of services, rather than financial constraints, as the primary barrier to accessing care.

**What are the implications for future research?**

Rural healthcare policy must continue to evolve. Efforts to attract and retain orthopaedic specialists to practice in rural West Virginia should be prioritized, and grants, along with other funding sources, should be made available to support rural outreach clinics to help prevent delays in orthopaedic care.

## Figures and Tables

**Table 1:** Demographics and Key Findings

Variable	Frequency (%)	Key Insights
Age ≥65	40%	Older patients cited transportation issues
Income < $30,000	55%	Financial strain compounded travel barriers
No bachelor’s degree	77%	Education level linked to delayed care
Reported Travel > 2 Hours	68%	Travel time a critical barrier
High Satisfaction w/ Care	85%	Positive experiences despite barriers

**Table 2:** Study Limitations

Limitation	Description
Sample Size	Small sample may limit generalizability
Lack of Pilot Testing	Non-validated tools could affect reliability
Single Geographic Focus	Findings specific to southern West Virginia counties
Cross-Sectional Design	Cannot assess changes over time
Self-Reported Data	Subject to recall bias
